# First report of an HIV-1 triple recombinant of subtypes B, C and F in Buenos Aires, Argentina

**DOI:** 10.1186/1742-4690-3-59

**Published:** 2006-09-07

**Authors:** María A Pando, Lindsay M Eyzaguirre, Marcela Segura, Christian T Bautista, Rubén Marone, Ana Ceballos, Silvia M Montano, José L Sánchez, Mercedes Weissenbacher, María M Ávila, Jean K Carr

**Affiliations:** 1 Centro Nacional de Referencia para el SIDA, Departamento de Microbiología, Parasitología e Inmunología, Facultad de Medicina, Universidad de Buenos Aires, Paraguay 2155, Piso 11, C1121ABG, Buenos Aires, Argentina; 2 Department of Epidemiology, Institute of Human Virology, University of Maryland Biotechnology Institute, 725 W. Lombard street, Baltimore, MD 21201, USA; 3 US Military HIV Research Program at the Walter Reed Army Institute of Research and the Henry M. Jackson Foundation for the Advancement of Military Medicine, Inc., 1 Taft Court, Suite 250, Rockville, MD 20850, USA; 4 Nexo Asociación Civil, Callao Av. 339, Piso 5, C1022AAD, Buenos Aires, Argentina; 5 US Naval Medical Research Center Detachment (NMRCD). Unit 3800, APO-AA 34031-3800 Lima, Peru; 6 Department of Defense Global Emerging Infections Surveillance and Response System (DoD-GEIS), Walter Reed Army Institute of Research, 503 Robert Grant Avenue, Room 1A30, Silver Spring, MD 20910, USA

## Abstract

We describe the genetic diversity of currently transmitted strains of HIV-1 in men who have sex with men (MSM) in Buenos Aires, Argentina between 2000 and 2004. Nearly full-length sequence analysis of 10 samples showed that 6 were subtype B, 3 were BF recombinant and 1 was a triple recombinant of subtypes B, C and F. The 3 BF recombinants were 3 different unique recombinant forms. Full genome analysis of one strain that was subtype F when sequenced in *pol *was found to be a triple recombinant. *Gag *and *pol *were predominantly subtype F, while gp120 was subtype B; there were regions of subtype C interspersed throughout. The young man infected with this strain reported multiple sexual partners and sero-converted between May and November of 2004. This study reported for the first time the full genome analysis of a triple recombinant between subtypes B, C and F, that combines in one virus the three most common subtypes in South America.

## Findings

The great genetic diversity of human immunodeficiency virus type 1 (HIV-1) strains have been recognized since early in the epidemic. Phylogenetic analyses of HIV-1 have revealed the presence of at least 9 subtypes (A-D, F-H, J and K) and 16 circulating recombinant forms (CRFs) worldwide [1].

In Argentina, previous molecular studies have revealed the presence of two epidemics; the first, among men who have sex with men (MSM), where the viral strains are mostly subtype B, and the second among heterosexuals and injecting drug users where BF recombinants predominate [2,3]. Further sequencing studies have also revealed the presence of a new CRF, the CRF12_BF [4] and subtype C [5]. These findings highlight the complex nature of the HIV epidemic in this country.

In this study, we performed nearly full-length genetic sequencing of 10 HIV-1 incident cases who seroconverted in a cohort study among MSM participants in Buenos Aires, Argentina, between the years 2002 and 2005 [6].

Only those subjects who were willing to participate and provided written informed consent were enrolled. Study participants provided clinic-epidemiologic data using a standardized questionnaire. A sample of anti-coagulated blood was collected in sterile fashion for peripheral blood mononuclear cells (PBMCs) separation. PBMC were separated by Ficoll-Hypaque and maintained at -70°C. PBMCs were used for DNA extraction by the QIAmp DNA extraction kit (QIAgen, Valencia, CA, USA). All samples were subjected to nearly full-length. PCR amplification using the Expand Long Template PCR System (Roche Applied Science, Penzberg, Germany) and a hot start method using a melting wax barrier (Dynawax). The first-round amplification was performed in a volume of 50 μl with primers MSF12b (5'-AAATCTCTAGCAGTGGCGCCCGAACAG-3') and OFMR1 (5'-TGAGGGATCTCTAGTTACCAGAGTC-3'). The second-round amplification was completed using 1μl of the first-round product and primers F2NST (5'-GCGGAGGCTAGAAGGAGAGAGATGG-3') and UNINEF 7 (5'-GCACTCAAGGCAAGCTTTATTGAGGCTTA-3'). This nested strategy amplifies about 9000kb of the HIV genome and was slightly modified from that used previously [7,8]. The amplified products were then sequenced with Big Dye terminators using an ABI 3100 automated sequencer (Applied Biosystems Inc, Foster City CA).

All sequences were assembled using the software Sequencher (Genecoes Inc., Ann Arbor MI) and examined in a multiple alignment with standard subtype references (Clustal X). Phylogenetic analyses were then conducted using Neighbor-joining method with Kimura's two-parameter model of distance calculation; bootstrap analysis was performed with 100 replicas. To study the ancestral relationships of triple recombinant sequence we conducted a Maximum Parsimony Analysis. Trees were constructed with the software PAUP version 4.0 (Sinaur Associates, Inc., Sunderland, MA) using tree-bisection-reconnection branch swapping (hold 10000) and bootstrap analysis (100 replicates).

Boostcan analysis and a visual inspection of the alignment were used to determine the presence of recombination and to locate breakpoints [7]. After the identification of the breakpoints, each segment was extracted and subjected to phylogenetic analysis to confirm the subtype assignment. Recombinant breakpoint locations were designated relative to HXB-2 (Genbank accession number: K03455).

**Figure 1:**
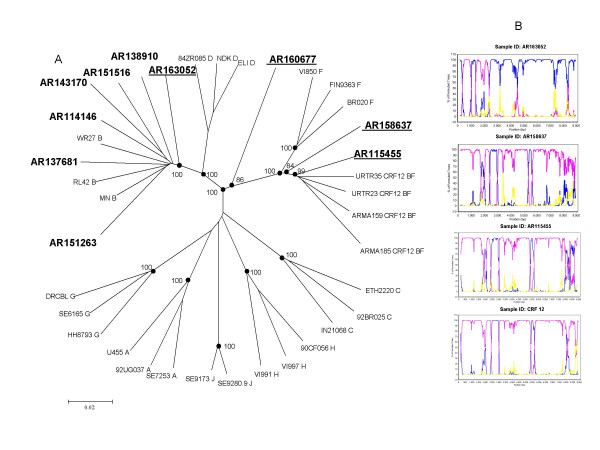
**Phylogenetic analysis of 10 nearly full-length sequences from acutely infected (seroincident) MSM participants in Buenos Aires, Argentina**. A) A neighbor-joining phylogenetic tree analysis was performed with the Kimura two-parameter method of distance estimation using reference sequences. The genetic distance corresponding to the lengths of the branches is shown by the bottom line. Studied samples are in bold. Underlined samples are inter-subtype recombinants. B) The bootscan analysis of nearly full length sequences of tree recombinant BF virus and CRF_12. This analysis was performed comparing the sample with subtype C (consensus of ETH2220, 92BR025 and IN21068), subtype B (consensus of WR27, MN and RL42) and subtype F (consensus of VI850, FIN9363 and BR020). A 300 nt window advanced in 20 nt increments was used.

**Figure 2:**
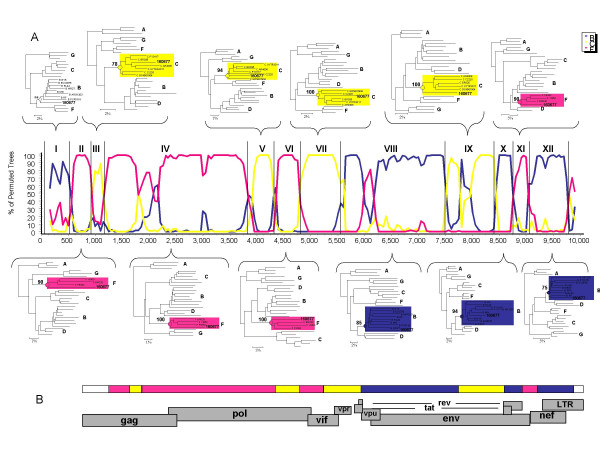
**Subtype structure and phylogenetic confirmation of the HIV-1 triple recombinant of subtypes B, C and F**. A) Bootscan analysis was performed comparing sample AR160677 to subtype C (consensus of ETH2220, 92BR025 and IN21068), subtype B (consensus of WR27, MN and RL42) and subtype F (consensus of VI850, FIN9363 and BR020). Neighbor-joining trees with bootstrap values were performed for each segment of the triple HIV-1 recombinant. A 300 nt window advanced in 20 nt increments was used. B) Diagram of the genes of HIV-1 shows the location of different genes across the triple recombinant.

Nearly full-length sequence analysis of 10 HIV-1 incident samples revealed that 6 of them were non-recombinant subtype B and 4 were recombinants (Figures 1a and 1b). Boostcan analysis showed that 3 of the 4 recombinants were BF recombinants (AR163052, AR158637, AR115455) and each of them had a unique recombination structure (Figure 1b). However, the recombinant sample AR160677 contained sequences corresponding to subtypes B, C and F (Figure 2).

The structure of this new BCF recombinant showed that g*ag *and *pol *were predominantly subtype F, while *env *was mostly subtype B; there were regions of subtype C interspersed throughout (Figures 2a and 2b). The subtype assignments of individual regions of this strain were confirmed in individual analysis shown in Figure 2a. Separate analysis confirmed the subtype assignments made by bootscan analysis; the only exception to this was the first segment, which could not be assigned with confidence to any subtype using any analytic techniques. This may be due to the presence of multiple short fragments of some or all of the 3 subtypes. In addition, maximum parsimony analysis suggested that the subtype C segments of this new recombinant had a common origin with subtype C strains from Brazil; however, no specific origin forsubtypes B or F could be detected (data not shown).

The recombinant sample belonged to a 26-year old man who reported having had multiple male sexual partners, including one from Brazil, and who seroconverted between May and November of 2004. This patient reported a syndrome compatible with primary HIV-1 infection [9] which included symptoms of fever, fatigue, myalgia, arthralgia, headaches, lymphadenopathy, nausea, vomiting, diarrhea, cough, anorexia, and weight loss of 5 kilograms (11 pounds) within 40 days of HIV diagnosis. The viral load was 64,050 copies per cubic milliliter (log 4.807) and the CD4 count was 391 cells per cubic milliliter. At that time the patient was negative for hepatitis B, hepatitis C and syphilis infections.

This study describes the first nearly full-length genome analysis of HIV-1 from MSM seroincident cases in Buenos Aires, Argentina. Subtype B was found to be the most common strain, however, three samples were found to be BF recombinants, and one sample was identified as a triple recombinant between subtypes B, C and F. Two of the BF recombinants shared some breakpoints with the CRF12_BF [4]; meanwhile, sample AR163052 showed a different pattern of recombination, suggesting that these samples originated via different recombination events.

Sample AR160677 represents the first triple recombinant of subtypes B, C and F identified in the region using nearly full-length genome sequencing. A report of a partial sequence of a BCF recombinant has been previously reported from southern Brazil [10], however, the subtype structure of that Brazilian recombinant is different from the structure of the triple recombinant being described.

We do not have additional patient information to determine if the HIV infection was due to a single infection with an already-circulating strain or it represented the result of two (or three) separate infections (i.e. superinfections) which ultimately lead to this unique strain as double and triple HIV infection has been previously described [12].

These recombinants have emerged in geographic areas where diverse HIV-1 genetic forms are co-circulating and where recombinant viruses are continually being generated in persons infected with two or more variants. Continued efforts to monitor the genetic makeup of HIV strains are critical in order to better assess the ongoing nature of the HIV epidemic in this region as illustrated by this case report.

## Abbreviations

HIV: Human Immunodeficiency Virus

CRFs: circulating recombinant forms

MSM: men who have sex with men

PBMCs: peripheral blood mononuclear cells

PCR: polymerase chain reaction

## Nucleotide accession number

GenBank accession numbers for the sequences of this study are [GenBank: DQ383746–DQ383755].

## Competing interests

The author(s) declare that they have no competing interest.

## Authors' contributions

MAP performed the laboratory work and wrote the manuscript with the help of LME. MS did the follow up of the cohort of MSM. CB helps in the data analysis and in the editing of the manuscript. RM designed and coordinated the field work at Nexo Asociación Civil. AC helps with the phylogenetic analyses. SMM and JLS made the international coordination of the cohort study. MMA directed the cohort study in Argentina with the help of MW. JKC designed and coordinated the study and the work at the laboratory. All the authors have read and approved the manuscript.
